# Circulating CEA‐positive and EpCAM‐negative tumor cells might be a predictive biomarker for recurrence in patients with gastric cancer

**DOI:** 10.1002/cam4.3616

**Published:** 2020-12-31

**Authors:** Yuichiro Miki, Masakazu Yashiro, Kenji Kuroda, Tomohisa Okuno, Shingo Togano, Go Masuda, Hiroaki Kasashima, Masaichi Ohira

**Affiliations:** ^1^ Department of Gastroenterological Surgery Osaka City University Graduate School of Medicine Osaka Japan; ^2^ Molecular Oncology and Therapeutics Osaka City University Graduate School of Medicine Osaka Japan

**Keywords:** CTC, EMT, gastric cancer

## Abstract

It has been reported that circulating tumor cells (CTCs) are beneficial for predicting tumor stage or treatment response. Although epithelial cell adhesion molecules (EpCAMs) and cytokeratin (CK) have been often used for the identification of CTCs, other tumor markers have not been fully investigated as detecting tools for CTCs. Thus, this study aims to clarify the significance of carcinoembryonic antigen (CEA, CD66e)‐positive CTCs in patients with gastric cancer. A total of 150 patients with gastric cancer were enrolled in this study. The mononuclear fraction of peripheral blood was enriched by Ficoll. The number of cells was enumerated depending on the positivity of EpCAM and CEA or CK by flow cytometry. The association of these cells with clinicopathologic characteristics was investigated. The mean age was 70 (range 28–92). The macroscopic type of gastric cancer was classified as 0/1/2/3/4/5 in 59/11/22/38/16/4 patients, respectively. Seventy‐one patients (47.3%) were diagnosed with intestinal‐type cancer, while 76 patients (50.7%) were diagnosed with the diffuse type. The mean numbers of cells with EpCAM−CK+, EpCAM+CK−, EpCAM+CK+, EpCAM−CEA+, EpCAM+CEA−, and EpCAM+CEA+ were 618, 237, 19.9, 1147, 291, and 7.41, respectively. The number of EpCAM−CEA+cells was significantly higher in patients with stage II–III and IV than in patients with stage I. The 3‐year RFS rate in patients with a high number of EpCAM−CEA+cells (>=622) was 57.5%, while it was 79.3% in patients with a low number of EpCAM−CEA+cells (<622) (log‐rank *p* = 0.0079). Thus, we conclude that CEA‐positive CTCs will be a clinically beneficial biomarker in patients with gastric cancer.

## Graphical abstract

This study aims to clarify the significance of epithelial cell adhesion molecules (EpCAMs) and carcinoembryonic antigen (CEA) expressing circulating tumor cells (CTCs) in patients with gastric cancer. The number of EpCAM−CEA+ cells was significantly associated with recurrence free survival. Thus, we conclude that CEA‐positive CTCs will be a clinically beneficial biomarker in patients with gastric cancer.

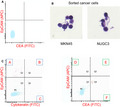

## 1 INTRODUCTION

Gastric cancer remains one of the most common malignancies and leading causes of cancer death worldwide.^1^, ^2^ Although the treatment outcomes have been improved, the recurrence rate after curative resection remains approximately 30% in patients with stage II–III gastric cancer.^3^ Thus, it is crucial to identify the patients who are at high risk for recurrence before an operation is performed.

Circulating tumor cells (CTCs), free cancer cells in the peripheral blood, could be a potential prognostic marker. To date, several clinical studies have proven the utility of CTCs.^4^, ^5^, ^6^, ^7^, ^8^, ^9^ For example, it has been reported that the detection and enumeration of CTCs were useful for monitoring treatment outcomes during chemotherapy.^7^ Okabe et al has reported that the CTC count performed using the CellSearch system was an independent prognostic factor in advanced gastric cancer patients.^9^ In many studies, the most common approach, including the CellSearch system, for identifying CTCs is the epithelial cell adhesion molecule (EpCAM)‐based enrichment technique.

CTCs are a heterogeneous population of cells, but the exact definition and established identification method of CTCs remain unclear. The epithelial–mesenchymal transition (EMT) is one of the cancer phenotypes that allow aggressive cancer cells to invade into blood vessels from the primary tumor. Since EpCAM is an epithelial marker, it might be difficult to identify CTCs with EMT using an EpCAM‐based technique.

In this study, we used carcinoembryonic antigen (CEA) for identifying CTCs including EMT cancer cells in patients with gastric cancer. We focused on the EpCAM‐negative (−), CEA‐positive (+) CTCs before the operation, and compared them with EpCAM+cytokeratin (CK)+ cell counts, since one of the most conventional definitions of CTCs is cells that are EpCAM+CK+. We evaluated the associations between the number of CTCs and clinicopathological factors and recurrence‐free survival (RFS). The aim of this study was to clarify the prognostic significance of EpCAM−CEA+ CTCs in patients with gastric cancer.

## 2 METHODS

### 2.1 Patients

A total of 150 patients with histologically proven gastric cancer between 2015 and 2019 were enrolled in this study. The pathological diagnoses and classifications were made according to the UICC TNM classification of malignant tumors seventh edition.^10^ The study protocol conformed to the ethical guidelines of the Declaration of Helsinki. This study was approved by the Osaka City University Ethics Committee (approval number 3159). Written informed consent was obtained from all patients.

### 2.2 Mononuclear cell (MNC) collection

Blood samples were collected from all patients preoperatively. The mononuclear cell (MNC) fraction was extracted according to the following protocol: 8 ml peripheral blood samples were carefully layered in BD Vacutainer CPT with heparin. The samples were spun in a centrifuge at room temperature for 15 min at 1800 *g*. Concentrated MNCs were harvested from the interface using a disposable pipette. As control, 8 ml peripheral blood samples were also collected from three healthy volunteers. We mixed two gastric cancer cell lines (MKN45 and NUGC3 cells) with these samples for confirming the capability of sorting cells.

### 2.3 Flow cytometric analysis

MNCs were stained with the following antihuman antibodies: Propidium Iodide Staining Solution (BD pharmingen, Catalog no. 556473), CD45‐BV421 (BD Horizon, Catalog no. 563879), CEA‐FITC (BIO‐RAD, Catalog no. MCA1744F), EpCAM‐APC (BD Biosciences, Catalog no. 347200), and Cytokeratin‐FITC (BD Biosciences, Catalog no. 347653). Labeled cells were analyzed using FACSAria^TM^ (BD). Dead cells were excluded by PI staining, and then, CD45 negative cells were analyzed by positivity of CEA, CK, and EpCAM. Isotype IgG antibody for anti‐CEA, anti‐EpCAM, and anti‐CK antibodies were used for control, and set the cutoff value for the positivity. We divided samples for each following staining; (a) CK and EpCAM, (b) CEA and EpCAM, and (c) isotype IgG. The number of cells for each fraction was recalculated to get the number of cells per 10 ml blood.

### 2.4 Statistical analysis

Associations between CTC counts and clinicopathological findings were analyzed using a *t*‐test. RFS was defined as the time from surgery to recurrence or death from any cause. RFS curves were estimated by the Kaplan–Meier method and compared using the log‐rank test. Univariate and multivariate analysis was performed using the Cox proportional hazards model. All statistical analyses were performed using JMP statistical software (version 8.0; SAS Institute). A two‐sided probability (*p*) value of <0.05 was considered to be statistically significant.

## 3 RESULTS

### 3.1 Patients

The patients’ backgrounds are summarized in Table 1. The mean age of the patients included was 70 (range 28–92). The number of male and female patients were 93 and 57, respectively. The macroscopic type of gastric cancer was classified as 0/1/2/3/4/5 in 59/11/22/38/16/4 patients, respectively. Seventy‐one patients (47.3%) were diagnosed with intestinal‐type cancer, while 76 patients (50.7%) were diagnosed with the diffuse type. This patient cohort included 16 (10.7) patients with Type 4 gastric cancer. Among 150 patients, 26 patients (19.3%) were diagnosed as Stage IV. Nineteen patients underwent staging laparoscopy, simple laparotomy (R2 resection), or bypass surgery, while 10 patients (6.6%) without non‐curative factors except CY1 underwent R1 resection by D2 lymphadenectomy, and 116 patients (77.3%) underwent R0 resection by gastrectomy with D1+ or D2 lymph node dissection. In total, 126 patients who underwent R0/1 resection were considered to be the cohort in which we could analyze the RFS.

**TABLE 1:** Patients’ background in 150 patients with gastric cancer

Variables	*n* (%) or median (range)
Age*	70 (28–92)
Sex	
Male	93 (62.0%)
Female	57 (38.0%)
BMI*	21.0 (13.6–31.3)
Main tumor location	
E	5 (6.2%)
U	24 (29.6%)
M	30 (37.0%)
L	22 (27.2%)
Macroscopic type	
0	59 (39.3%)
1	11 (7.3%)
2	22 (14.7%)
3	38 (25.3%)
4	16 (10.7%)
5	4 (2.7%)
Histology	
Intestinal	71 (47.3%)
Diffuse	76 (50.7%)
Others	3 (2.0%)
Stage	
I	68 (45.3%)
II	27 (18.0%)
III	29 (19.3%)
IV	26 (17.3%)
Operation	
Total gastrectomy	41 (27.3%)
Distal gastrectomy	84 (56.0%)
Proximal gastrectomy	6 (4.0%)
Staging laparoscopy, simple laparotomy, and bypass surgery	19 (12.7%)
Lymph node dissection	
D0	4 (3.0%)
D1	23 (17.2%)
D1+	56 (41.8%)
D2	51 (38.0%)

Values in parentheses are percentages unless indicated otherwise.

* Median (range).

### 3.2 Relationship between CTC counts and clinicopathological factors in gastric cancer

**FIGURE 1:**
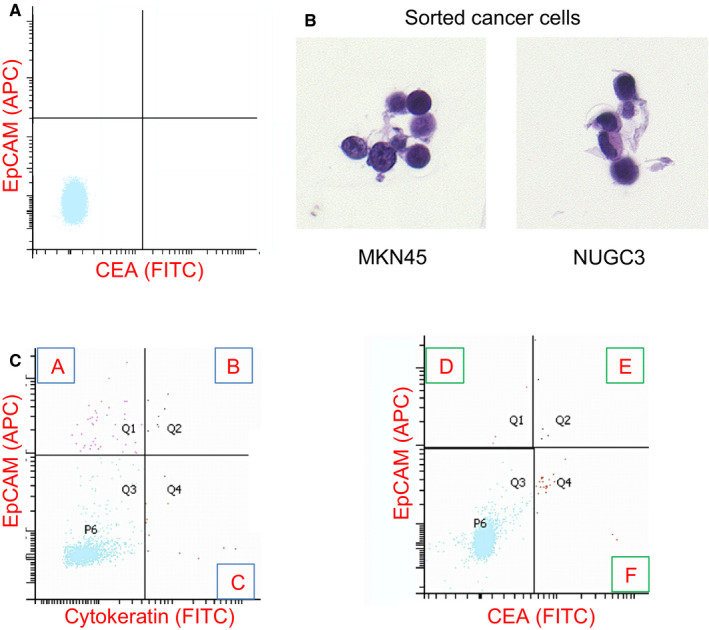
(A) Representative pictures showing flow cytometry results using EpCAM and CEA from healthy volunteer. (B) Gastric cancer cells sorted by FACS. (C) Representative pictures showing flow cytometry results using cytokeratin (left) or CEA (right) and EpCAM. The number of cells were enumerated depending on the following six fractions. (A) EpCAM+CK−, (B) EpCAM+CK+, (C) EpCAM−CK+, (D) EpCAM+CEA−, (E) EpCAM+CEA+, and (F) EpCAM−CEA+

Representative results of the flow cytometry analysis are shown in Figure 1. On the analyses from three healthy volunteers, all CD45 negative cells did not express either EpCAM, CK, or CEA (see the representative analysis; Figure 1A). After spiking the gastric cancer cell lines to the sample from healthy volunteer, MKN45 and NUGC3 cells were sorted by EpCAM positivity. The morphology of these sorted cells are shown in Figure 1B.

**FIGURE 2:**
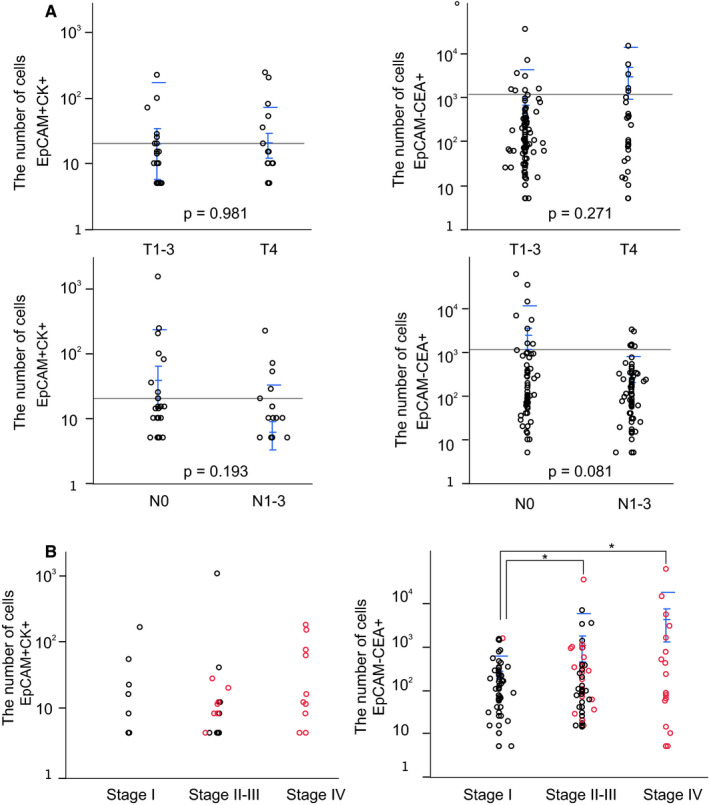
(A) Correlations between T and N factors and EpCAM+CK+ cells (left) and EpCAM−CEA+ cells (right). (B) Correlations between stage and EpCAM+CK+ cells (left) and EpCAM−CEA+ cells (right). Blue bar shows the mean and standard deviation of each group, and black bar shows the mean of all patients

Among propidium Iodide (PI) staining negative, CD45 negative cells, the mean counts of EpCAM−CEA+, EpCAM+CEA−, and EpCAM +CEA+ cells were 1147, 291, and 7.41 cells/10 ml total blood, respectively (see the representative analysis; Figure 1C). As a result of the same analysis in which CK was used as a CTC marker, the mean counts of EpCAM−CK+, EpCAM+CK−, and EpCAM+CK+ were 618, 237, and 19.9 cells/10 ml total blood, respectively (see the representative analysis; Figure 1C). The number of EpCAM−CEA+ cells was significantly higher in patients with Stage IV than in patients with Stage I–III (*p* = 0.011, Table 2). The numbers of EpCAM−CK+ and EpCAM−CEA+ cells were significantly associated with resection status (R0/1 or not, Table 2). The numbers of EpCAM+CK+ cells and EpCAM−CEA+ CTCs cells per patient are plotted in Figure 2A. These cell numbers are not significantly associated with T stage, N stage, tumor size, serum CEA, or CA19‐9 (data not shown). The number of EpCAM+CK+ cells or EpCAM−CEA+ cells per patient was plotted depending on stage, and recurrent cases are shown as red circles in Figure 2B. There were no significant differences among stages I, II–III, and IV regarding the number of EpCAM+CK+ cells. On the contrary, the number of EpCAM−CEA+ cells was significantly higher in stage II–III and stage IV than in stage I.

**TABLE 2:** The number of circulating cells in each fraction depending on Stage or R state

	Total	Stage I–III	Stage IV	*p*‐value	R0–1	Others	*p*‐value
EpCAM−CK+	618 (4001)	378.7 (363.7)	1768.6 (796.1)	0.114	182.7 (351.8)	2928.9 (810.3)	0.0023
EpCAM+CK−	237 (678)	236.3 (62.1)	243.4 (136.0)	0.961	234.8 (61.6)	251.8 (141.9)	0.912
EpCAM+CK+	19.9 (128)	18.1 (11.7)	28.4 (25.7)	0.714	19.2 (11.6)	23.2 (26.8)	0.891
EpCAM−CEA+	1147 (6062)	616.7 (538.3)	4228.8 (1297.5)	0.011	622.4 (541.5)	4033.6 (1269.9)	0.014
EpCAM+CEA−	291 (1356)	311.1 (123.1)	177.8 (296.8)	0.679	209.0 (122.4)	745.3 (287.2)	0.088
EpCAM+CEA+	7.41 (60.1)	7.5 (5.4)	6.9 (13.1)	0.966	1.70 (5.55)	38.7 (12.5)	0.0075

Values in parentheses are standard deviation unless indicated otherwise

### 3.3 RFS

**FIGURE 3:**
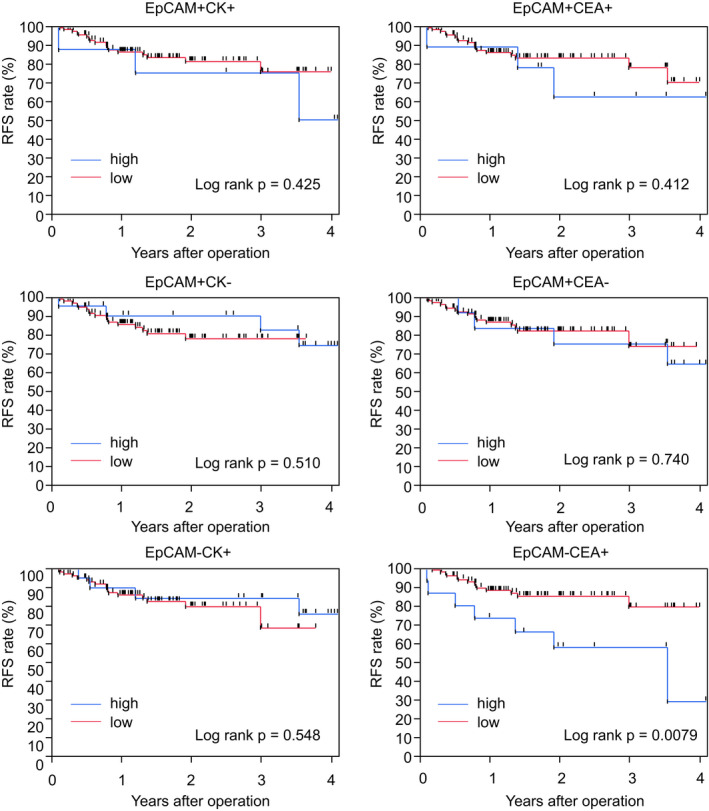
Recurrence‐free survival using six different fractions of circulating cells

As for survival analyses, we set the mean number of cells for each fraction of circulating cells as the cutoff value in patients who underwent R0/1 resection. Among six types of fractions, only EpCAM−CEA+ cells showed significant survival impact (log‐rank *p* = 0.0079, Figure 3). The 3‐year RFS rate in patients with a high number of EpCAM−CEA+ cells (>=622) was 57.5%, while it was 79.3% in patients with a low number of EpCAM−CEA+ cells (<622).

Multivariate analysis was performed to validate the significance of EpCAM−CEA+ CTCs. Among the seven covariates shown in Table 3, the EpCAM−CEA+ cell number as well as lymph node metastasis were shown to be independent significant prognostic factors in patients with gastric cancer.

**TABLE 3:** Univariate and multivariate analyses with respect to recurrence‐free survival after surgery in 126 patients with gastric cancer who underwent R0/1 resection.

	Univariate		Multivariate	
	Odds ratio (95% CI)	*p*‐value	Odds ratio (95% CI)	*p*‐value
EPCAM−CEA+ cells count (high)	3.21 (1.21–7.76)	0.020	3.87 (1.00–15.0)	0.049
Age (≥ 70 y.o.)	1.47 (0.65–3.53)	0.349	2.80 (0.91–9.61)	0.071
Histologic type (Undifferentiated)	1.53 (0.68–3.55)	0.299	1.29 (0.37–4.35)	0.68
Tumor depth (T4)	6.86 (2.97–15.6)	<0.001	3.00 (0.84–10.4)	0.087
Lymph node metastasis (Positive)	9.93 (3.75–34.4)	<0.001	7.63 (2.04–37.0)	0.0025
Lymphatic invasion (2–3)	2.93 (1.08–7.39)	0.035	2.32 (0.64–9.52)	0.21
Venous invasion (2–3)	1.51 (0.23–5.32)	0.600	1.73 (1.02–11.6)	0.46

## 4 DISCUSSION

Liquid biopsies to detect CTCs offer a unique opportunity to find potential biomarkers associated with cancer progression. We here revealed that circulating EpCAM−CEA+ cells were significantly associated with poor survival in patients with gastric cancer. Although several studies have demonstrated that the enumeration of CTCs is useful for predicting survival, most previous studies have focused on EpCAM+ and/or CK+ cells. Thus, to the best of our knowledge, this is the first study in which the significance of EpCAM−CEA+ circulating cells is shown.

To date, EpCAM and CK have been often used to identify CTCs. However, since both of them are considered as epithelial markers, any combination of these markers` positivity cannot designate the cell fraction which shows EMT. Thus, we used CEA for the identification of cancer cells in this study.

We were able to detect CEA+ circulating cells and also enumerate these cells in gastric cancer patients. CEA is one of the cell adhesion factors first identified in human colon cancer tissues. CEA is still one of the most often and most routinely used tumor markers in clinical settings for patients with gastric cancer, and it is known that more than 90% of patients with gastric cancer express CEA in their tissues.^11^ Previously, a polymerase chain reaction‐based method was used for detecting CEA expression in circulating cells.^12^, ^13^, ^14^ Importantly, flow cytometry could enumerate the number of CEA+ cells. As cancer cells express CEA with high percentage, CD45−CEA+ cells should include cells derived from cancer tissue. Among them, CD45−CEA+EpCAM− cells might include cancer cells with EMT, because EpCAM is epithelial marker. Considering that the number of CEA+ cells and serum CEA protein concentration were not statistically correlated, secreting CEA and cell‐surface CEA may play different roles in tumor progression.

Surprisingly, EpCAM−CEA+ cells had the strongest prognostic impact among the six fractions of circulating cells we tested in this study. We consider that this fraction should be related to the EMT phenotype of CTCs. EMT has been demonstrated to be an important step in the formation of metastasis. During EMT, cells change from an epithelial phenotype, with tight adhesions and polarized layers, to a mesenchymal phenotype, without polarization. Although many researchers have stated the heterogeneity of CTCs,^8^, ^15^ tumor cells should undergo EMT before entering into blood circulation, so it should be quite reasonable that the number of cells in this kind of mesenchymal CTC fraction has a more powerful prognostic value than the number in the other fractions. It will be interesting to check the expression of EMT markers, such as vimentin, N‐cadherin, etc., in EpCAM−CEA+ CTCs, although it still remains unclear which marker is the best for identifying EMT‐like CTCs. However, we consider that the simple enumeration of EpCAM−CEA+ cells is one of the most convenient ways to identify EMT‐like CTCs.

The enumeration of CTC including EpCAM−CEA+ cells may be beneficial to select patients who undergo preoperative chemotherapy (neoadjuvant chemotherapy; NAC), because our data shows that preoperative EpCAM−CEA+ cell counts has prognostic value. Preoperative chemotherapy for patients with gastric cancer have not been considered as standard treatment for gastric cancer. JCOG0501 study could not show the efficacy of NAC for patients with type4 and large type3 gastric cancer. However, the clinical trial to evaluate the efficacy of perioperative chemotherapy (NAC+ Adjuvant) is ongoing (JCOG1711), we need to seriously consider the way to select appropriate patients for NAC by using several clinical markers including CTCs data.

Recently, Li et al have reported that chromosome aneuploidy contribute to the phenotypic evolution of HER2 expression on CTCs after HER2 targeted therapy.^16^ They used HER2‐iFISH technology, in which in situ hybridization and immunostaining of CTC markers can be simultaneously performed. They also used the automated scanning system, and defined target HER2 positive CTCs as DAPI+, CD45‐, HER2+, with diploid or aneuploid Chromosome 8. Considering that the number of chromosome affects the positivity of CTC marker, iFISH method could be important in future.

As has been described, CTC detection methods are controversial. Furthermore, CTCs should be heterogeneous, and it remains unknown what kind of subpopulation is the best for the prediction of survival and the monitoring of treatment response. A well‐designed prospective study with a larger sample size should be undertaken in the future to answer these questions. In this study, we counted the number of CTCs just before the operation, but counting the cells during the treatment course after the operation or during chemotherapy may be beneficial for clinical decision making, so this matter should also be evaluated in a future study.

In conclusion, CEA‐positive and EpCAM‐negative CTCs might be a predictive biomarker for recurrence in patients with gastric cancer.

## CONFLICT OF INTEREST

There are not any financial or other interests with regard to the submitted manuscript that might be construed as a conflict of interest.

## Data Availability

The data that support the findings of this study are available from the corresponding author upon reasonable request.
